# Pulmonary Endothelial Mechanical Sensing and Signaling, a Story of Focal Adhesions and Integrins in Ventilator Induced Lung Injury

**DOI:** 10.3389/fphys.2019.00511

**Published:** 2019-04-26

**Authors:** Gabriel T. Kelly, Reem Faraj, Yao Zhang, Emin Maltepe, Jeffrey R. Fineman, Stephen M. Black, Ting Wang

**Affiliations:** ^1^Department of Internal Medicine, College of Medicine Phoenix, The University of Arizona, Phoenix, AZ, United States; ^2^Department of Pediatrics, University of California, San Francisco, San Francisco, CA, United States; ^3^Department of Medicine, College of Medicine, The University of Arizona, Tucson, AZ, United States

**Keywords:** ARDS, VILI, focal adhesion, mechanical stress, cyclic stretch, integrin β4

## Abstract

Patients with critical illness such as acute lung injury often undergo mechanical ventilation in the intensive care unit. Though lifesaving in many instances, mechanical ventilation often results in ventilator induced lung injury (VILI), characterized by overdistension of lung tissue leading to release of edemagenic agents, which further damage the lung and contribute to the mortality and progression of pulmonary inflammation. The endothelium is particularly sensitive, as VILI associated mechanical stress results in endothelial cytoskeletal rearrangement, stress fiber formation, and integrity loss. At the heart of these changes are integrin tethered focal adhesions (FAs) which participate in mechanosensing, structure, and signaling. Here, we present the known roles of FA proteins including c-Src, talin, FAK, paxillin, vinculin, and integrins in the sensing and response to cyclic stretch and VILI associated stress. Attention is given to how stretch is propagated from the extracellular matrix through integrins to talin and other FA proteins, as well as signaling cascades that include FA proteins, leading to stress fiber formation and other cellular responses. This unifying picture of FAs aids our understanding in an effort to prevent and treat VILI.

## Introduction

Ventilator induced lung injury (VILI) is a clinical syndrome in the intensive care unit that results from mechanical ventilation. It is often associated with overdistension as well as vascular leak caused by edemagenic agents and inflammatory cytokines such as thrombin, histamine, tumor necrosis factor-α, interleukin-8, and interleukin-1 (Dos Santos and Slutsky, 2000; Lionetti et al., 2005; Birukova et al., 2006). VILI associated mechanical stress imposes severe pro-inflammatory lung endothelial injury, leading to endothelial integrity loss, cytokine secretion, and vascular leakage. It is well believed that the focal adhesion (FA)-integrin system, as the bridge between endothelial and basal matrix, serves as a principal mechanical stress sensing and transducing complex. This review will focus on the known roles of various FA proteins in endothelial cells (ECs) in response to VILI associated mechanical stress, specifically cyclic stretch (CS).

## Endothelial Injury in VILI

### VILI and Endothelial Injury

Acute lung injury (ALI) and its more severe form Acute Respiratory Distress Syndrome (ARDS) are devastating conditions with an unacceptable mortality of approximately 40% about 2 weeks after the onset of the syndrome (Villar et al., 2011). The recent “Berlin” definition defines ARDS as an acute primary pulmonary condition characterized by radiologic infiltrates and impaired oxygenation (Laffey and Kavanagh, 2017). This condition may be the result of any number of underlying causes including direct lung injury such as pneumonia, aspiration, or traumatic pulmonary contusion, or indirect injuries such as non-pulmonary sepsis or non-septic shock (Frutos-Vivar et al., 2004). These underlying causes lead to inflammatory, ischemic, mechanical, or infective insults on the lung, triggering damage to alveolar capillaries, interstitium, and epithelium, which leads to increased vascular permeability and results in subsequent interstitial and alveolar edema (Laffey and Kavanagh, 2017). Mechanical ventilation is one of the lifesaving strategies for ARDS, yet the mortality rate of ARDS patients remains high with ventilation associated persistent lung inflammatory injury, which is called Ventilator-Induced Lung Injury or VILI (Slutsky and Ranieri, 2014).

Similar to ARDS itself, VILI can induce a range of inflammatory responses such as increases in lung vascular permeability due to damage to the endothelial cell barrier and subsequent alveolar flooding. The development and course of VILI is associated with mechanical ventilator settings including dose and pattern (Gajic et al., 2005). Similar to ARDS, VILI directly leads to damages to the gas exchange barrier or complete dysfunction of alveoli leading to respiratory failure in patients (Villar et al., 2014). Particularly, mechanical ventilation increases in the alveolar epithelial cell surface area by 1/3 (Tschumperlin and Margulies, 1999), with a similar effect in capillary endothelium which forms tight contacts with alveolar epithelium. This longitudinal tension produced by the mechanical ventilator also induces various cellular responses including mechanical stress associated molecular signaling, ROS generation, gene expression, and cellular remodeling (Birukov, 2009), leading to damage directly to ECs, which can be observed at the ultrastructural level (Dreyfuss and Saumon, 1998). This persistent VILI associated mechanical stress during ventilation leads to further dysregulation of the pulmonary capillary endothelium, leading to protein rich fluid leakage from the capillaries to the interstitium and continuing into the alveoli, resulting in life-threatening pulmonary edema (Cruz et al., 2018). Once lung damage occurs, lung ECs express pro-inflammatory cytokines and signaling molecules to further exacerbate vascular permeability, vascular tone, leukocyte recruitment, and apoptosis (Villar et al., 2014).

### Endothelial Mechanical Sensing in VILI

Ample evidence has been found to support that reorganization of the pulmonary endothelial cytoskeleton caused by mechanical stress leads to VILI (Lionetti et al., 2005). Many ARDS studies also suggest that transient receptor potential (TRP) channels are activated in lung injury induced by mechanical stress, and some certain types of TRP, including TRPV4, facilitate mechanical stress sensing (Parker et al., 1998; Alvarez et al., 2006). This is complicated, however, by the fact that these responses are post-cellular injury, and particularly since other cellular stresses including heat, osmolarity changes, and metabolites can also activate TRP channels in a similar pattern (Darby et al., 2016; Simonsen et al., 2017). Given the fact that mechanical stress originates from the misalignment between the basal membrane and the cytoplasmic membrane, linker complexes, including FAs, between the two parties, must be the first line mechanical stress sensors for the cells to initiate other cellular responses (Geiger and Bershadsky, 2002). Interestingly, FAs are also dynamic regulators of cytoskeletal remodeling, where assembly, disassembly, and structure alteration adjust the formation and displacement of actin fibers (Oakes and Gardel, 2014).

### Endothelial Cell Cyclic Stretch Experimental Models

Measurements of mechanical stress in the mechanically ventilated lung are technically challenging due to the complexity of local distension patterns in the lung parenchyma, however, calculations have been made to suggest that if the lung volume increases by 40% of the total lung capacity, the alveolar epithelial cell basal surface area increases by 34% (Tschumperlin and Margulies, 1999; Tschumperlin et al., 2000; Wirtz and Dobbs, 2000). High tidal volume mechanical ventilation results in a 40–50% surface area increase as would be reflected *in vitro* by 18% CS or repeated stretch (Tschumperlin et al., 2000; Birukov, 2011), and spontaneous breathing with a 25% surface area increase can be reflected as 5% CS (Tschumperlin et al., 2000; Wirtz and Dobbs, 2000; Birukov, 2011). Here 18 and 5% CS is the measure of the cell length elongation in one dimension compared to resting conditions, although the ECs may be exposed to one or two dimensional stretch (Wang J.H. et al., 2001). CS is accomplished by growing monolayers of ECs to confluence on flexible membranes and stretching those membranes either on commercial systems (Wang et al., 2000; Colombo et al., 2008) or custom machines. Published *in vitro* experiments of VILI mainly use 10–20% CS with 0.4–2 Hz of frequency to reflect the 20–120 breaths per minute ventilation used in the intensive care unit (Rimensberger, 2003; Belteki and Morley, 2018). In this review, in order to clarify the different experimental outcomes with different mechanical stretch conditions, amplitude of stretch (% CS) and frequency are noted in each *in vitro* research data cited.

## Focal Adhesions in VILI

### Endothelial FA

**FIGURE 1:**
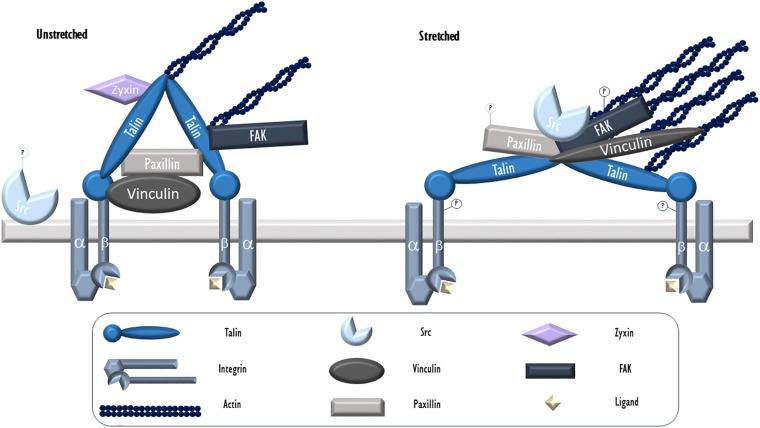
Endothelial focal adhesion in cyclic stretch. Integrins, heterodimers consisting of α and β subunits, serve as the key physical link between FAs and the ECM. During VILI, mechanical stress (cyclic stretch) causes calcium release from intracellular stores and c-Src activation via dephosphorylation. It then localizes to patches along the cytoskeleton and FAs where it targets and phosphorylates FA proteins including integrin β, paxillin, and FAK. Phosphorylated and activated FAK facilitates the formation of stress fibers. Talin is an adaptor protein essential for integrin connection to the cytoskeleton. In its activated form, talin dimers are bound to actin and likely assume a Y-shape with exposed vinculin binding sites. Vinculin is a cytosolic actin-binding protein that exists in a circular configuration localized close to integrins and binds paxillin. Upon activation, vinculin assumes an extended form and moves further toward actin fibers. Paxillin, another adaptor FA protein activated by c-Src mediated phosphorylation following stretch, allows for protein networking and signal transduction. Zyxin, a LIM protein, resides at unstretched FAs but dissociates and moves away from FAs and toward stress fibers during stretch. -P, phosphorylated.

Focal adhesions (FAs) may be described as discrete areas on a cell’s basal surface located at the end of “stress fibers,” prominent bundles of actin filaments, which contain integrins and a variety of associated proteins. These provide anchor points for cells to adhere to their substratum, as well as sense and transmit forces and biochemical signals between cells and matrix (Wu, 2005). Essential to FAs are integrins, transmembrane proteins that bind matrix ligands extracellularly and a series of structural proteins that link it to the cytoskeleton intracellularly. These structural proteins include talin, focal adhesion kinase, paxillin, tensin, filamin, and α-actinin, as well as many other associated linkers and signaling proteins (Figure 1). To date, over 900 proteins have been found in FAs (Kuo et al., 2011). In ECs, FAs play central roles in angiogenesis, wound healing, vascular remodeling, cytoskeletal arrangement, and barrier regulation (Wu, 2005).

During mechanical ventilation or related cellular stretch, like all other cell types, lung ECs respond to mechanical forces largely through the action of the actin cytoskeleton. Mechanical force associated cellular signals, often deleterious, rely on the contractile activity of F-actin associated actomyosin networks- interconnected two-dimensional contractile meshworks that include actomyosin fibers and their anchor points (Lecuit et al., 2011). This is especially true during mechanical ventilation when no pathologically high mechanical stress by blood flow (often seen in pulmonary hypertension or other pulmonary vascular diseases) is present. Located in association with the ECM, FA complexes are the main participants in ECs to receive mechanical stimuli and serves as key mechanical tension sensing and signaling hubs through complex signaling events such as post-translational modifications, binding to cytoskeletal proteins or kinases, and structural changes (Zaidel-Bar and Geiger, 2010). They play a central role in receiving and transducing mechanical stress to the cytoplasm, via associated transmembrane integrins (Figure 1).

### SRC Proto-Oncogene, Non-receptor Tyrosine Kinase

One of the key components and the most critical kinase in FAs is cellular Src (c-Src), the prototypical member of the Src family of kinases (Kefalas et al., 1995). Under normal unstressed conditions, N-terminal myristoylation causes c-Src association with plasma membranes, but relocation occurs via cytoskeletal trafficking (Jones et al., 2000). C-Src has kinase activity to phosphorylate its substrates, including autophosphorylation on Y^416^ (Cooper and MacAuley, 1988). C-Src interacts with these targets (or other binding partners) through its SH2 and SH3 domains (Cooper and MacAuley, 1988). c-Src tertiary structure and activity can be regulated by the phosphorylation state of a tyrosine residue (Y^527^) at the C-terminal (Cooper and MacAuley, 1988). Phosphorylation at this site by c-terminal Src kinase (Csk) inactivates c-Src, while dephosphorylation by calcineurin, a Ca^2+^r/calmodulin-dependent protein phosphatase (PPIIB), activates it (Cooper and King, 1986).

During VILI, c-Src is activated through a variety of means. Mechanical stretching of ECs leads to Ca^2+^ influx through the opening of stretch-activated cation channels (SA channels), which in turn initiates Ca^2+^ release from intracellular calcium storage (Adams et al., 1989; Naruse and Sokabe, 1993; Naruse et al., 1998c). Histamine, whose levels rise in inflammatory lung injuries (Kim et al., 2005), also triggers [Ca^2+^] oscillations (Adams et al., 1989). This rise in intracellular [Ca^2+^] or [Ca^2+^]_i_ activates c-Src, giving rise to many of the morphological changes seen in ECs during CS (Naruse et al., 1998a). Once c-Src is dephosphorylated and activated, it localizes to patches along cytoskeletal structures, as well as FAs where it targets several FA proteins including integrins (Hirst et al., 1986), FAK, and paxillin (Sokabe et al., 1997). Inhibition of c-Src activation or kinase activity prevents most of the effects of CS including cell alignment and formation of stress fibers that occurs with 20% CS at 1 Hz (Naruse et al., 1998a) and disassembly of adherans junctions that occurs with 18% CS at 0.4 Hz (Tian et al., 2016). C-Src activation subsequent to activation of VEGF receptor 2 (VEGFR2) leads to phosphorylation of cytoskeletal proteins outside of FAs by c-Src when subjected to 18% CS at 0.4 Hz for 120 min (Tian et al., 2016). Some of the effects of these phosphorylation events will be discussed later.

### Talin

Talin is found universally in FAs and in fact is the only essential adaptor protein for integrin connection to the cytoskeleton (Jiang et al., 2003). Evolutionally, it is older than integrins, leading some to consider talin as the master of FAs (Klapholz and Brown, 2017). Talin is structurally split into a head domain, a short linker domain, and a long rod domain (Klapholz and Brown, 2017). The N-terminal FERM domain forms the head and is split into four subdomains termed F0-F3. F0 binds Rap1 small GTPase proteins. F1, F2, and F3 can bind directly to membranes. F2 and F3 can bind to actin via the actin binding site 1 (ABS1). The F3 domain can bind to the cytoplasmic domain of β integrin subunits at its integrin binding site 1 (IBS1) which is a variant of the canonical phosphotyrosine binding (PTB) domain (Garcia-Alvarez et al., 2003). F3 also can interact with many associated proteins including FAK (Klapholz and Brown, 2017). The rod domain is comprised of subunits R1-R13, and contains multiple sites for protein–protein interactions. Actin binding site 2 (ABS2) is located at R3-R8 and ABS3 is located at R13. The integrin binding site 2 (IBS2) falls within R11, and consists of two five-helix bundles connected by a kinked continuous helix (Gingras et al., 2009). Activation of integrin is required for this binding since the binding site on β integrin is on the same face that binds the tail of α integrin (Rodius et al., 2008). At least 18 vinculin binding sites have been found in the rod domain (Gingras et al., 2005). In addition, more binding sites for Rap1-GTP-interacting adaptor molecule (RIAM), paxillin, α-synemin, deleted in liver cancer 1 (DLC1), and KN motif and ankyrin repeat domain proteins Kank1 and Kank2 have been found within the rod domain. Talin’s only known activities are through binding other proteins, making it a classic adapter protein (Klapholz and Brown, 2017).

When bound to actin, talin is likely in its dimer form (Goldmann et al., 1994) and adopts a Y-shape (Winkler et al., 1997) or dumbbell shape (Hemmings et al., 1996). The dimerization domain is at the C-terminus, and just upstream are linker regions thought to be flexible. Molecular dynamics simulation in the Mofrad lab has shown that one of talin’s vinculin-binding sites (VBS1) is inactive unless pulled open by a stretching force (Lee et al., 2007). Cellular stretching causes hydrophobic residues on the VBS1 surface to rotate around a neighboring alpha-helix, exposing the hydrophobic vinculin binding pocket. This was shown to operate *in vitro* and was reversible (Yao et al., 2016). The same research group (Mofrad) that performed the molecular dynamics went on to confirm that the distance between integrins bound by a talin dimer alters the angle of the talin dimerization regions (Golji and Mofrad, 2014). The varying distance between integrins that occurs during stretching of the basement membrane during VILI makes this a likely method of mechanosensing within FAs. The stretch of talin modeled above is converted into a cellular response by vinculin (Elosegui-Artola et al., 2016).

Aside from stretch related stress, chemical factors released during VILI are capable of exerting effects on talin through vinculin. Although details have not been clarified, an active F-actin binding site on vinculin is required for production of thrombin induced talin positive FAs to cause increased endothelial monolayer permeability (Birukova et al., 2016).

### Vinculin

Vinculin is a cytosolic actin-binding protein that is involved in stabilizing actin polymerization and recruiting actin remodeling proteins (Bays and DeMali, 2017). Like talin, it has no enzymatic activity. Its structure consists of a large head domain, a short linker, and a tail. When inactive in the cytosol, vinculin is in a circular configuration where the head and tail have a very tight bond, inhibiting interactions with other proteins. But when activated, such as in FAs, vinculin exists in an extended form (Chen et al., 2005). Many proposals have been presented on just how vinculin is opened, including multiple ligand interaction (Bois et al., 2006; Chen et al., 2006), single ligand displacement (Izard et al., 2004), phosphorylation (Golji et al., 2012), and stretching (Golji and Mofrad, 2010).

Vinculin requires a level of tension to remain in a FA and prevent disassembly of FAs, making it a likely mechanosensor by itself (Carisey et al., 2013). When inactive, vinculin is localized closer to integrins and binds paxillin. When activated by binding to talin, vinculin is seen to move further away from integrin where it interacts strongly with actin (Case et al., 2015). This seems to paint a picture where stretch induced FA rearrangement includes opening of vinculin so that it can stabilize stress fibers.

### Focal Adhesion Kinase

Focal Adhesion Kinase (FAK) is an adapter protein, as well as an active kinase found at FAs and other locations throughout the cell. It is activated both through recruitment to FAs following integrin activation as well as by phosphorylation. Multiple tyrosine, serine, and threonine phosphorylation sites have been mapped by mass spectrometry (Grigera et al., 2005).

Exposure of human pulmonary ECs to 18% CS at 25 cycles per minute leads to FAK phosphorylation at Y^397^ and Y^576^ (Shikata et al., 2005). This CS also induces FAK re-distribution to the ends of newly formed stress fibers. Similar results were seen in bovine ECs. Besides phosphorylation and rearrangement of FAK, paxillin is phosphorylated and rearranges, events which were dependent on Rho/p21 activity (Yano et al., 1996a,b). Interestingly, some of these changes are mediated by SA channel opening and c-Src activation, while SA channel inhibition does not eliminate the changes to FAK and paxillin, suggesting other activation/inactivation mechanisms besides c-Src are at play (Sawada and Sheetz, 2002).

The pathophysiological changes caused by VILI are not mediated exclusively by tissue overdistension (by volume increase) or high frequency ventilation (by frequency increase). Agents such as thrombin and cytokines are also found at high levels in VILI patients and contribute to the pathology of this injury. Thrombin binds to its receptor PAR-1, thereby mediating FAK redistribution patterns similar to stretch (Shikata et al., 2003a). This is mediated by phosphorylation of the same residues (Y^397^ and Y^576^) as those seen in stretch but also at Y^925^. By contrast, c-Src phosphorylation of FAK at Y^576^ only is sufficient to cause many of the barrier protective effects seen in sphingosine-1-phosphate mediated endothelial barrier protection including peripheral translocation of FA proteins and cortical actin ring formation (Shikata et al., 2003a). This demonstrates the varying signaling pathways that can be activated by the phosphorylation profile of FAK.

When cells are cyclically stretched in only one direction, stress fibers form perpendicularly to the stretch direction. This alignment is dependent on the type of stretch applied. For example, when bovine aortic ECs are subjected to 10% CS, actin alignment is increased proportionally to the frequency (0.01 to 1 Hz) of CS (Shirinsky et al., 1989; Sokabe et al., 1997; Hsu et al., 2010). The involvement of FAK in stress fiber formation as a result of CS is controversial. Some reports show FAK phosphorylation, which occurred during 20% CS at 1 Hz, is required for this process (Sokabe et al., 1997), while others show that FAK/paxillin knockdown in ECs or overexpression in fibroblasts do not block stress fiber formation at 10% CS at 1 Hz (Hsu et al., 2010; Ngu et al., 2010).

More evidences have been generated to prove the role of FAK in endothelial signaling activated by mechanical stress. Uniaxial 10% CS at a rate of 3 cycles per minute activates cell proliferation in ECs (Sumpio et al., 1987). Additionally, FAK activation plays a role in cell proliferation in a variety of cell types including epithelial and fibroblasts. In epithelial cells, 10% CS at 20 cycles per minute activates Y^418^ phosphorylation of c-Src as well as the two tyrosine sites on FAK already mentioned (Y^397^ and Y^576^). These lead to downstream ERK1/2 activation and proliferation within 5 min after initiation of CS (Wang J.G. et al., 2001; Chaturvedi et al., 2007). Fibroblasts stretched 20% at 1 Hz activate the same pathway, though FAK is phosphorylated at Y^397^ and Y^925^ (Wang J.G. et al., 2001). Further light on the pathway of proliferation was gleaned in osteoblast-like cells where CS activated FAK, c-Src, and proline-rich tyrosine kinase 2 (PYK2). Although c-Src was not found to be necessary, FAK associated with PYK2 and led to ERK2 phosphorylation mediated by the Ras/Raf/MEK pathway (Boutahar et al., 2004). It is logical that the same pathway would function in pulmonary endothelia since 10% CS at 1 Hz activates ERK1/2 phosphorylation in bovine ECs, though the p21ras/PI3K pathway may also be involved (Ikeda et al., 1999). This PYK2 phosphorylation is mediated by c-Src (Cheng et al., 2002). Also, it appears that 12% CS at 1 Hz results in Ca^2+^ dependent PKCα activation leading to NADPH oxidase activity in ECs to produce ROS. This ROS is crucial for c-Src and PYK2 activation. Interestingly, pulmonary injury induced by 20% CS at 0.5 Hz-causes a reduction of FAK phosphorylation and activity following a transient increase in phosphorylation at 30 min of injury (Desai et al., 2009). This emphasizes the damaging effects of VILI on an already injured lung.

As discussed above, VILI leads to an increase in pulmonary vascular permeability. FAK plays a critical role in both initiation and integrating signaling pathways that regulate barrier function (Wu, 2005), though whether FAK causes barrier loss or protection is unclear. For example, in mice with ECs expressing defective FAK, pulmonary vascular permeability was severely compromised, an abnormal distribution of vascular endothelial cadherin (VE-cadherin) was observed, and reduced VE-cadherin Y^658^ phosphorylation was measured (Zhao et al., 2010). Additionally, FAK knockdown inhibits the normal barrier enhancement provided by sphongosine-1-phosphate (Zhao et al., 2009). On the other hand, FAK knockdown in pulmonary ECs exhibits a number of barrier enhanced phenomenon including stronger cell-cell contacts and a greater number and size of vinculin plaques (Arnold et al., 2013). All these data suggest that FAK is a key FA molecule regulating endothelial injury and signaling upon mechanical stress.

### Paxillin

Paxillin is another adapter protein of FAs that allows for protein networking and signal transduction. Phosphorylation occurs primarily at Y^31^ and Y^118^ by FAK or Src family kinases (Schaller and Parsons, 1995) but also is targeted at Y^40^, Y^88^, and Y^181^ (Nakamura et al., 2000; Schaller and Schaefer, 2001). Five LD domains (LD1-5) near the N-terminus have been identified that function as protein interaction interfaces for actopaxin, ILK, vinculin, papillomavirus E6, FAK/PYK2, the Arf-GAPs p95PKL/GIT2/GIT1, and some evidence shows PAK3, clathrin, and PABP1 (Brown and Turner, 2004). Paxillin directly binds integrin β (Schaller et al., 1995).

During 10% CS at 1 Hz, paxillin levels remain unchanged, but phosphorylation is induced (Yano et al., 1996a). Whereas paxillin will exhibit a speckled pattern in static conditions, strain causes paxillin to align to the long axis of cells in parallel to F-actin. Inhibition of tyrosine phosphorylation blocks paxillin rearrangement, F-actin alignment, and cell elongation. This inhibition of tyrosine phosphorylation was indiscriminate for paxillin and FAK, so distinct roles for FAK versus paxillin could not be delineated. It is also interesting that cell migration is mediated in part by paxillin/FAK phosphorylation, and this is proportional to the degree of stretch. The upstream activator of phosphorylation in this case is not verified, but it is highly possible that activated c-Src (Sokabe et al., 1997) phosphorylates paxillin and leads to cytoskeletal rearrangement. Inhibition of FAK phosphorylation also prevents paxillin phosphorylation at 20% CS 1 Hz, demonstrating that paxillin phosphorylation occurs downstream of FAK activation (Naruse et al., 1998b).

Paxillin is not required for maintenance of a FA. In ECs, 20% CS at 0.5 Hz induced paxillin rearrangement is transient (Huang et al., 2012). Additionally, this study showed that paxillin only plays a role in the early portion of FA rearrangement; paxillin knockdown inhibited FA formation at 10 min CS, but did not inhibit FA formation at 30 min or longer.

### Other FA Proteins

G protein-coupled receptor kinase-interacting target 1 (GIT1) has been proposed to be involved in FA disassembly (Zhao et al., 2000; Shikata et al., 2003b; van Nieuw Amerongen et al., 2004). GIT1 under normal conditions exhibits a cytoplasmic distribution. In ECs, some GIT1 is found weakly distributed to the end of stress fibers at FA following 18% CS (Shikata et al., 2005) and thrombin exposure (van Nieuw Amerongen et al., 2004) where it colocalizes with FAK and vinculin. RhoA and Rho kinase are required for this recruitment, providing evidence that the signal for GIT1 is derived outside of the FA. At the FA, GIT1 is phosphorylated either by Rho kinase or c-Src, and contributes to stretch or thrombin induced cell rounding and contraction, FA formation, and FAK phosphorylation.

Zyxin is a LIM protein that resides at FAs (Beckerle, 1997). In a number of cell types including umbilical vein ECs, under 15% CS at 0.5 Hz, zyxin moves away from FAs and associates with stress fibers (Yoshigi et al., 2005). The trigger for movement is derived at least in part by integrin activation and not by SA channels. The only known role of zyxin in these conditions is to reinforce the actin structure by organizing thicker filaments.

## Integrins in VILI

**FIGURE 2:**
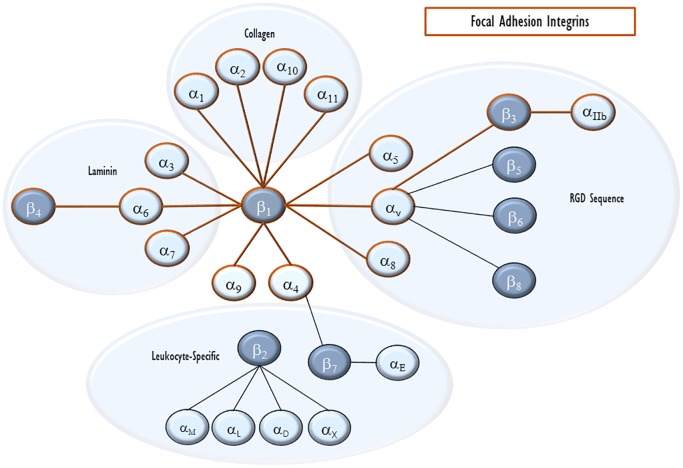
Integrin pairing. Integrins are heterodimeric cell-surface receptors for ECM proteins. Each heterodimer consists of an α and a β subunit. The eighteen α integrins and eight β integrins are shown with lines connecting pairs that are able to heterodimerize with each other. They are also clustered by their major ligand. Integrins that can enter endothelial focal adhesions are outlined in red.

Integrins are heterodimer transmembrane proteins that serve as the key physical anchor for the FA to the cytoplasmic membrane and the connection between FA and the ECM. They consist of an α and a β subunit. There are now eighteen identified α subunits and eight β subunits. Each subunit contains extracellular, transmembrane, and cytoplasmic domains. Based on the exact pairing, extracellularly, integrins bind particular ECM components such as collagen, laminin, fibronectin, and vitronectin, as well as some other cellular receptors and signals including E-cadherin, prothrombin, and von Willebrand factor (Plow et al., 2000; Figure 2). Intracellularly, β chains alone seem to define cytoskeletal interactions (Pan et al., 2016). Because of this, we will focus on the roles of β integrins with known roles in VILI, mentioning their binding pair when known.

β integrins, with the exception of β4, contain short cytoplasmic sequences (40–60 amino acids) (Sastry and Horwitz, 1993). Among these, the sequences of β1-3 and 5–7 are very similar. Adjacent to the transmembrane domain is a short sequence of 11 mostly charged amino acids. The second cluster contains a NPIY sequence that can be phosphorylated by c-Src and similar kinases (Hirst et al., 1986). An NPXY sequence comes next, though the spacing is variable between different β integrins. All three sequences, at least in integrin β1, are needed for full integration into FAs.

Because of their structure and location, integrins can mediate signaling in two directions (Hu and Luo, 2013). Binding of extracellular matrix proteins leads integrin heterodimers to undergo a conformational change including separation of cytoplasmic tails of the two integrin subunits, allowing for interactions between these tails and cytoplasmic proteins and thus propagating signals (Luo et al., 2007; Zhu et al., 2007). This has been termed outside-in signaling. Conversely, binding of intracellular proteins such as talin or kindlin result in integrin subunit separation (Anthis et al., 2009; Ye et al., 2011). This separation alters structural conformation such that the extracellular domains have an increased affinity for ligands (Hu and Luo, 2013). This has been termed inside-out signaling. β integrins in FAs are already bound to talin, FAK, and paxillin, which prevent association between subunits, keeping the integrin in an open conformation (Kim et al., 2011; Hu and Luo, 2013). It is imaginable, however, that during distension of the ECM during stretch, inactive integrins may come into contact with extracellular ligands leading to outside-in signaling and formation of new FAs. In our discussions of talin and vinculin, we saw that some of the events involved in FA activation during CS are dependent upon propagation of force from the ECM to these proteins. Obviously integrins, as the link across the plasma membrane, are central to this propagation (Geiger et al., 2009; Hu and Luo, 2013). This force propagation may be considered another outside-in mechanosensing function of integrins within the FA.

In most research on FAs, the exact integrin involved is not identified. In FAs of ECs, the most recognized β integrins are β1 and β3. It is also worth note that these two integrins are often interchangeable in which types of FAs they incorporate (Sastry and Horwitz, 1993).

Many of the studies used to analyze the role of integrins took little into account with regard to available ECM proteins and conditions. Since different αβ pairs have different ECM protein binding substrates, results of experiments can be misleading when a particular integrin is being measured and its substrate is not available on the culture surface (Hirayama and Sumpio, 2007). Detailed experiments have even showed that integrin heterodimer pairs switch depending on the availability of extracellular cations (Stuiver et al., 1996).

### Integrin β1

Integrin β1 is referenced most often in the literature of FAs. It can be phosphorylated at T^777^, Y^783^, S^785^, T^788^, T^789^, and Y^795^, but only requires phosphorylation of T^788^ or T^789^ to be active (Wennerberg et al., 1998; Nilsson et al., 2006). This phosphorylation is achieved by PKCε (Stawowy et al., 2005).

Upon 10% CS at 1 Hz, integrin β1 redistributes to the ends of stress fibers, similar to other FA proteins FAK or paxillin. In capillary ECs, static stretch (15% elongation) induces integrin β1 phosphorylation at T^788^/T^789^ within 1 min of force application (Thodeti et al., 2009). Inhibition of T^788^/T^789^ phosphorylation prevents strain-induced cell reorientation, stress fiber alignment, and redistribution of FAs (Sastry and Horwitz, 1993; Hu and Luo, 2013). Upstream of phosphorylation was opening of the SA channel TRPV4 followed by PI3K activity.

One aspect not often studied with regard to CS is the influence of neighboring cells. One study addressed this by stretching isolated cells stretch (Huang et al., 2011). They noted that under static conditions, integrin β1 is loosely distributed throughout ECs. Following 20% stretch activation of confluent cells at a frequency of 0.5 Hz, integrin β1 levels rise and clustering is observed in lines perpendicular to the direction of stretch after 10 min. In isolated cells, however, integrin β1 failed to cluster or align in this fashion, showing that intercellular junctions play a role in endothelial cell cytoskeleton rearrangement and not just focal adhesion mechanosensing. Another report also recognized the redistribution of integrin β1 upon stretch to linear patterns, but did not see a rise in β1 levels at the mRNA level (Yano et al., 1997). The binding partner for integrin β1 is dependent on the coating of the stretched surface; when coated with fibronectin, the fibronectin receptor integrin α5β1 is found, and when coated with collagen, the collagen receptor α2β1 is found.

### Integrin β3

Integrin β3 is a receptor for vitronectin whether dimerized with integrin αv or αIIb. Umbilical vein ECs exposed to 20% CS at a frequency of 1 Hz demonstrate significantly elevated integrin β3 levels of mRNA after 4 h and elevated protein expression at 12 h (Suzuki et al., 1997). Equally, the number of FAs containing integrin β3 increased. These results are debatable, however, because in similar experiments (at the less strenuous conditions of 10% CS at 1 Hz) using the same type of cells, integrin β3 did not follow FA rearrangement (Yano et al., 1997).

We have seen above that thrombin levels are elevated in VILI, and that thrombin regulates FAK and GIT1 distribution. Some evidence shows a secondary effect of thrombin to induce angiogenesis in ECs by directly binding to integrin αvβ3 (Tsopanoglou et al., 2002). However, these effects are overshadowed by the damage caused by overdistension and probably only plays a role during healthy physiologic breathing (Birukova et al., 2006).

### Integrin β4

Integrin β4, with a very different cytoplasmic domain compared to other β integrins, does not incorporate into traditional FAs. Nevertheless, integrin β4 interacts with a number of FA proteins. And as we shall see, it has the most complex reaction to VILI of all the β integrins.

The only known partner for integrin β4 is integrin α6 (Hynes, 2002). Integrin α6β4’s extracellular target is laminin-5. Unlike other FA β integrins, β4’s cytoplasmic tail interacts with intermediate filaments through plectin. Syndecan-1, a cell surface proteoglycan which plays roles in FA structure, can bind integrin β4 as well as other intracellular and extracellular components (Altemeier et al., 2012; Wang et al., 2014).

The cytoplasmic tail of integrin β4 is 1088 amino acids long. This tail is composed of a proximal Calx Na-Ca exchanger domain followed by two pairs of fibronectin type II repeats with a tyrosine activation motif (TAM) between them (Hogervorst et al., 1990). Several tyrosines and serines can be phosphorylated; some of the tyrosines are targeted by Src family kinases (SFKs) including Fyn and Yes (Giancotti, 2007), and serines are targeted by epidermal growth factor (EGF) and/or PKCα (Rabinovitz et al., 2004). Phosphorylation by these kinases results in binding of FAK to integrin β4 (Tai et al., 2015). The exact point of interaction seems to be just proximal to the transmembrane domain of integrin β4 to a sequence of FAK just upstream of its Y^397^ autophosphorylation site (amino acids 376–386).

Integrin β4 is tyrosine phosphorylated on at least one of Y^1440^, Y^1526^, Y^1640^, or Y^1422^ within 30 min of exposure to 18% CS at 0.5 Hz (Chen et al., 2015). This phosphorylation is required for the full effects of CS induced inflammatory factor release into media, though it seems that at least one phosphotyrosine also provides protective effects. In a broader model, it seems that integrin β4’s cytoplasmic tail is responsible for many of the effects of VILI, as mutant mice lacking the cytoplasmic portion are almost completely protected from high tidal volume ventilation. Integrin β4 binding to laminin-5 does not seem to be required for the morphological changes seen in stretched ECs when other integrins are available (Hirayama and Sumpio, 2007).

### Other Integrin β

Of the five remaining β integrins, β5 is the only one that is significantly expressed in the pulmonary endothelium, though data supporting localization within FAs is lacking (Wayner et al., 1991; Sastry and Horwitz, 1993). There is evidence, however, that integrin αvβ5 associates with FAK from c-Src activity in developing ECs (Eliceiri et al., 2002). This integrin certainly plays a role in VILI progression because knockout mice are protected from VILI and blocking integrin αvβ5 *in vitro* prevents thrombin injury (Su et al., 2007). And integrin β5 signaling is required for stretch induced changes in epithelial cells and associates with zyxin (Bianchi-Smiraglia et al., 2013). Integrin β6 is heavily concentrated in epithelium but is not normally expressed in endothelium (Tabata et al., 2008). Integrins β2 and β7 are principally expressed in leukocytes, but β7 is also found in some endothelia where it heterodimerizes with α4 and responds to a number of inflammatory agents (Brezinschek et al., 1996). Integrin β8, with a non-classical cytoplasmic domain compared to β1-3 and β5-7, was originally characterized as unimportant with regard to adhesion or the cytoskeleton (Nishimura et al., 1994). Later, however, it was found in ECs where it plays a role in angiogenesis (Giusti et al., 2013). Evidences of the involvement of these other types of integrin βs in FAs are very limited and require further experiments.

## Summary and Conclusion

Ventilator induced lung injury is characterized by compromised vascular endothelial barrier protection and the production of edemagenic agents in response to mechanical stretch that may lead to overdistention depending upon tidal volume and ventilation frequency (Dos Santos and Slutsky, 2000; Lionetti et al., 2005; Birukova et al., 2006). This mechanical-force initiated cellular injury results in cytoskeletal rearrangement. FAs play a central role in mechanotransduction and cytoskeletal rearrangement (De et al., 2010; Ladoux and Nicolas, 2012; Iskratsch et al., 2014; De, 2018). Here, we present the known picture of the molecular changes that occur at FAs as a result of CS and other factors of VILI. Within the FA, force from stretching of the ECM is propagated through integrins into the FA (Geiger et al., 2009). This force is then exerted on adapter and signaling proteins such as talin and vinculin. Additionally, other receptors for stretch and agents involved in VILI result in phosphorylation events on c-Src and then on integrin, FAK, paxillin, and others. Together these lead to the characteristic stress fiber formation and cytoskeletal rearrangement seen in VILI.

The most essential proteins within the FA are integrins and talin (Jiang et al., 2003). These are the minimum structural components which link the ECM to the actin cytoskeleton. Talin may be considered the master regulator of FAs (Klapholz and Brown, 2017), but integrins anchor the FA to the plasma membrane, are the first proteins involved in a newly formed FA (Romer et al., 2006) and are the first link in the outside-in function of FA force propagation.

Ventilator induced lung injury remains a substantial health care burden with an obvious lack in therapies (Dos Santos and Slutsky, 2000; Plataki and Hubmayr, 2010). More research on the nature of VILI progression is essential in order to prevent and treat patients with this injury. This review focusses on individual FA proteins currently known to be involved in VILI related pathology. By integrating this knowledge with other VILI research on the cellular, tissue, organ, and individual level, it is hoped that new prevention and treatment methods will continue to reduce the impact of VILI. By investigating each individual protein as we have, we can better understand the basic mechanisms of mechanical stress on endothelial injury. This should help us identify more specific targets for drug therapies in VILI. Biological agents which target many of these proteins such as c-Src, FAK, paxillin, and integrins have been developed in other disease models (Paulhe et al., 2005; Infusino and Jacobson, 2012; Eke and Cordes, 2015), though currently none are approved for VILI therapy. The possibility that these agents would prove efficacious in VILI is difficult to determine because of the incomplete knowledge we have on the disease. Additionally, the various mathematical, *in vitro*, animal, and clinical data lead to many contradicting findings regarding the roles of individual proteins in VILI. Further research and understanding of individual proteins and sites within proteins should help develop a “more pure” understanding of the roles these proteins play and allow for more selective targeting and drug development.

More advanced tools are being developed to study VILI and related disease. One such tool is genetics. It is already common practice to personalize mechanical ventilation strategies using a variety of monitoring inputs (Nieman et al., 2017). Additionally, genetic influences may be at play, as health disparities are common in pulmonary injury (Moss and Mannino, 2002; Frutos-Vivar et al., 2006; Erickson et al., 2009), and genetic variants have been found to play a role in VILI and other pulmonary diseases (Barnes, 2005; Nonas et al., 2005; Gao et al., 2006). We are sure that variants in FA proteins will continue to be found and their function evaluated.

In summary, the FA-integrin complex is a key mechanical stress biosensor system in ECs in response to VILI. Although complex and sometimes controversial, the FA-integrin system modulates VILI associated endothelial injury and signaling, and obviously is a viable drug target for VILI. Our perspective is that the next breakthrough in FA-integrin research is the genetic influences on variable VILI outcome, which will lead to a better understanding of the pathobiology of endothelial mechanical stress sensing and signal transduction, thus more importantly providing a basis for personalized medicine.

## Author Contributions

All authors wrote the text and edited the figures.

## Conflict of Interest Statement

The authors declare that the research was conducted in the absence of any commercial or financial relationships that could be construed as a potential conflict of interest.
